# A Quantitative Assessment of the Role of the Parasite *Amoebophrya* in the Termination of *Alexandrium fundyense* Blooms within a Small Coastal Embayment

**DOI:** 10.1371/journal.pone.0081150

**Published:** 2013-12-04

**Authors:** Lourdes Velo-Suárez, Michael L. Brosnahan, Donald M. Anderson, Dennis J. McGillicuddy

**Affiliations:** 1 Department of Applied Ocean Physics and Engineering, Woods Hole Oceanographic Institution, Woods Hole, Massachusetts, United States of America; 2 Department Dynamiques de l’Environnement Côtier, Institut Français de Recherche pour l'Exploitation de la MER, Plouzané, France; 3 Department of Biology, Woods Hole Oceanographic Institution, Woods Hole, Massachusetts, United States of America; CSIR- National institute of oceanography, India

## Abstract

Parasitic dinoflagellates of the genus *Amoebophrya* infect free-living dinoflagellates, some of which can cause harmful algal blooms (HABs). High prevalence of *Amoebophrya* spp. has been linked to the decline of some HABs in marine systems. The objective of this study was to evaluate the impact of *Amoebophrya* spp. on the dynamics of dinoflagellate blooms in Salt Pond (MA, USA), particularly the harmful species *Alexandrium fundyense*. The abundance of *Amoebophrya* life stages was estimated 3–7 days per week through the full duration of an annual *A. fundyense* bloom using fluorescence in situ hybridization coupled with tyramide signal amplification (FISH- TSA). More than 20 potential hosts were recorded including *Dinophysis* spp., *Protoperidinium* spp. and *Gonyaulax* spp., but the only dinoflagellate cells infected by *Amoebophrya* spp. during the sampling period were *A. fundyense*. Maximum *A. fundyense* concentration co-occurred with an increase of infected hosts, followed by a massive release of *Amoebophrya* dinospores in the water column. On average, *Amoebophrya* spp. infected and killed ∼30% of the *A. fundyense* population per day in the end phase of the bloom. The decline of the host *A. fundyense* population coincided with a dramatic life-cycle transition from vegetative division to sexual fusion. This transition occurred after maximum infected host concentrations and before peak infection percentages were observed, suggesting that most *A. fundyense* escaped parasite infection through sexual fusion. The results of this work highlight the importance of high frequency sampling of both parasite and host populations to accurately assess the impact of parasites on natural plankton assemblages.

## Introduction

Parasitism is a common interspecific interaction in aquatic habitats. Quantitative understanding of the mechanisms of parasitism has improved rapidly in the past decade and there is increasing evidence that parasites can exert significant influence on marine ecosystems through impacts to host population dynamics and interruption of interspecific interactions. In many systems, parasites are important drivers of biodiversity and exert significant influence on the flow of energy through microplanktonic and microbial food webs [1].

*Amoebophrya* spp. belong to Syndiales (Alveolata) and are a widespread group of marine parasites [2]. *Amoebophrya* spp. infect and kill a taxonomically broad variety of dinoflagellates, including harmful algal bloom (HAB) species within the genera *Alexandrium*, *Dinophysis*, *Karlodinium* and *Akashiwo* [3]. During their life-cycle, *Amoebophrya* alternates between a free-swimming infective stage (dinospore) and a multinuclear growth phase (trophont) within its host [4], [5]. An infection starts when a dinospore attaches to a host cell’s outer surface, then enters the host’s cytosol. Once inside, the parasitoid can either remain in the cytoplasm or invade the host’s nucleus where it transforms into a trophont. The trophont increases in size while undergoing a series of nuclear divisions and flagellar replications without completing cytokinesis. Late-infection trophonts are large, multinucleate and multiflagellate, occupying most of the host cell and have a characteristic “beehive” appearance [6]. Once fully matured, the trophont expands through the cell wall of the host and transforms into a strongly motile vermiform stage. The vermiform is short-lived, soon separating into many individual infective dinospores. Dinospores must find a new host soon since their survival time in the water column is short (∼10 days) [7].

Infection of dinoflagellates by *Amoebophrya* spp. has received particular interest in recent years, at least in part due to the potential of parasitism to exert top-down control of HABs [8]–[11]. Previous field studies have shown that *Amoebophrya* spp. infection success can be moderate to high (20%–80%), with the highest infection percentages occurring near the termination of host blooms [9], [11]. High host concentrations have been cited as a pre-condition for high infection rates [3], but *Amoebophrya* infections may also be a significant cause of host mortality at low host concentrations [12]–[14]. Under certain conditions, *Amoebophrya* infections may sometimes have a greater impact on the population dynamics of bloom-forming dinoflagellates than grazing by microzooplankton, and can potentially eliminate an entire host population within a few days [15], [16]. Still, there are significant gaps in our knowledge of the interactions between *Amoebophrya* spp. and their hosts, including the in situ host specificity of different *Amoebophrya* strains.

Recent advances in molecular techniques to study picoplankton communities have provided unprecedented tools to study the abundance and diversity of the *Amoebophrya* free-living dinospores and early-stage infections in their natural environment [11], [17]. The ability to detect and quantify free-living *Amoebophrya* spp. dinospores has increased our knowledge considerably and allows the understanding of *Amoebophrya* spp. infectivity and the characterization of parasite-host dynamics in natural assemblages. In this study we applied fluorescence *in situ* hybridization coupled with tyramide signal amplification (FISH- TSA) with an oligonucleotide probe for *Amoebophrya*. We use this method to examine the distribution and abundance of free-living *Amoebophrya* dinospores and estimate the abundance of infected dinoflagellate hosts in Salt Pond (Eastham, MA USA), a drowned kettle pond within the Nauset Marsh system that experiences annual shellfishing closures due Paralytic Shellfish Poisoning (PSP). These PSP events are caused by the HAB species *Alexandrium tamarense* Group I, which we refer to hereafter as *A. fundyense*, a renaming that has been proposed by U. John and others ([18]).

Salt Pond is an ideal study site because *Amoebophrya* spp. have long been known to infect *A. fundyense* there [19] and because host *A. fundyense* blooms are selectively retained within the pond due to an interaction between the pond’s circulation, bathymetry, and *A. fundyense*’s swimming behavior [20]. These aspects of the study site enabled us to plan our sampling using *A. fundyense* as a focal host species because changes in *A. fundyense* abundance could be directly attributed to growth, mortality and sexual processes without considering advection of the host population in and out of the system. This study also coincided with the first ever deployment of an Imaging FlowCytobot (IFCB) in Salt Pond. The IFCB is an *in situ* imaging-in-flow cytometer that records images of phytoplankton 10–100 µm in length, including *A. fundyense* cells [21]. The instrument provided near real-time estimates of *A. fundyense* abundance and also a detailed record of the *A. fundyense* population’s transition to the sexual phase of its life cycle [22].

The primary objective of this study was to assess the quantitative importance of *Amoebophrya* parasitism to *A. fundyense* bloom termination in Salt Pond.

## Materials and Methods

### 2.1 Study Area

**Figure 1:**
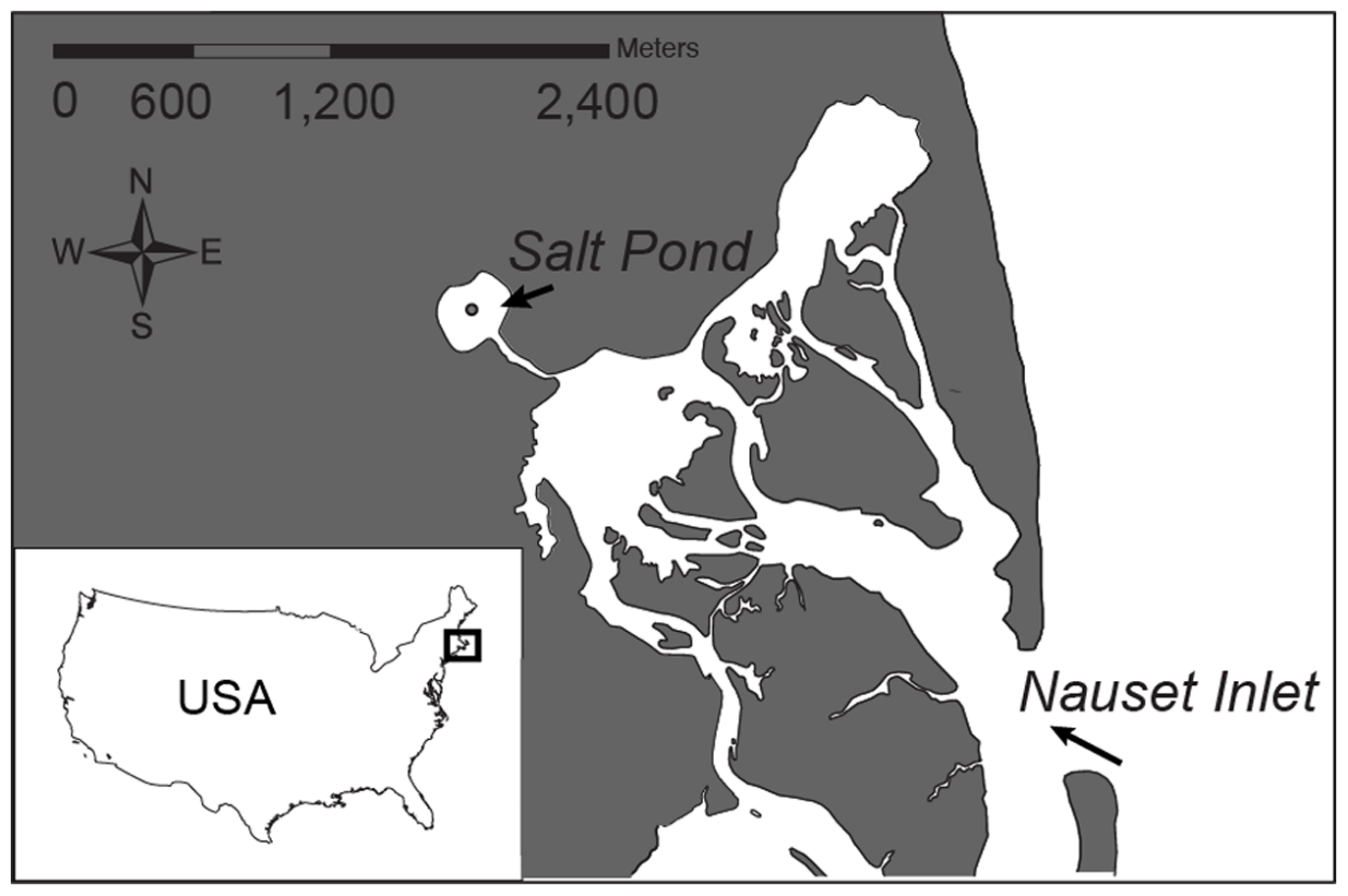
Nauset Marsh System map. Map of the study area showing the location of Salt Pond inside the Nauset Marsh System. Station 21 in the center of the Pond is marked in grey.

Salt Pond is a saline pond at the northwestern boundary of the Nauset Marsh system (NMS; Eastham, MA USA; Fig. 1). The NMS is a shallow estuary with extensive marshes protected from the Atlantic Ocean by a highly dynamic barrier beach with a single connection through Nauset Inlet [23]. Salt Pond is roughly circular, with a surface area of 82,200 m^2^, an average depth of 3.4 m, and a maximum depth of 9 m [20]. It is connected to the Nauset Marsh by a narrow inlet channel 0.5 km long, 30 m wide and 0.5 m deep at low tide. The pond receives freshwater input from groundwater but there are no direct stream or river inputs. Tidal currents are weaker and stratification due to salinity and temperature gradients are stronger than in the shallower central marsh.

### 2.2 Sampling Overview

Authors were issued a permit from the United States Department of the Interior, National Park Service, Cape Cod National Seashore to conduct survey work throughout the Nauset Marsh including Salt Pond in Eastham, MA for the years from 2010 to 2015. The title of the overarching project is “Toxic Alexandrium blooms in the Nauset Marsh System”, led by Dr. Donald Anderson (Permit ID: CACO-2010-SCI-0001).

Field collections were taken at 1–3 day intervals from a float near the center of Salt Pond, from early March to mid May 2012 (Fig. 1). Whole seawater samples of 200 mL were collected at 1, 3 and 5 m depth using a 2.5 L Niskin bottle. Additional samples were taken at 7 m depth on 27 and 29 Apr 2013. Samples were immediately fixed on board with formaldehyde (1% final concentration) and stored on ice. Once in the laboratory, samples were prescreened through a 100 µm pore size net to remove large zooplankton and divided into two size-fractions (<15 µm and 15–100 µm) with a 15 m sieve. Both size fractions were gently filtered (100 mm Hg) onto polycarbonate filters (Millipore, 25 mm diameter; 0.8 µm pore size for smaller fraction; 5 µm pore size for larger fraction) to collect Amoebophryidae dinospores (<15 µm) and the microplankton community (15–100 µm) separately. Filters were dehydrated in a series of ethanol washes (50, 80 and 100%, 3 min each), dried at room temperature and stored at −20°C until analysis (typically within 3 months of collection). On the day of analysis, filters were thawed at room temperature and then cut into pieces with a razor blade. To avoid cell loss, filter sections were dipped in low-gelling-point agarose gel (0.2%), dried face down on Parafilm, and then dehydrated with ethanol.

### 2.3 Enumeration of A. fundyense

*A. fundyense* abundances were quantified using whole cell fluorescence *in situ* hybridization (FISH) using the oligonucleotide probe NA1 (5′-AGT GCA ACA CTC CCA CCA-3′) conjugated to a Cy3® fluorochrome [24], [25]. Briefly, a 1/4 piece of the 5 µm filters was immersed in 1 mL of pre-hybridization buffer and incubated at RT for 5 minutes. Filters were removed from the pre-hybridization solution and immersed in 1 mL of hybridization buffer containing the probe. Hybridization was performed at 50°C for 1 hour in an aluminum foil wrapped microcentrifuge tube temperature block (Eppendorf NA). Filters were washed at 50°C for 5 min with 1 mL of freshly made washing buffer.

After hybridization, filters were mounted on a microscope slide and stored at 4°C in the dark until analysis. The entire filter section was counted using a Zeiss Axioskop epifluorescence microscope equipped with a 10X and 20X coupled with a Cy3® fluorescence filter set (Chroma #41032).

### 2.4 Amoebophrya spp. Prevalence and Dinospore Abundance

Fluorescence *in situ* hybridization coupled with tyramide signal amplification (FISH-TSA) was used to enumerate (1) infected dinoflagellate host cells, with special emphasis on *A. fundyense* and (2) free living *Amoebophrya* spp. dinospores. A horseradish peroxidase (HRP) conjugated, *Amoebophrya* genus-specific oligonucleotide probe (ALV01; 5′-GCC TGC CGT GAA CAC TCT-3′) was used because it has been tested to work well on different strains of *Amoebophrya* in both culture and field samples [11], [26] and targets most of the environmental sequences belonging to Amoebophyidae. (68% of 1970 sequences) [11].

The FISH-TSA procedure was adapted for the identification of infected dinoflagellate host cells and estimation of *Amoebophrya* spp. prevalence (% of infected dinoflagellate hosts) [13], [27]. One eighth sections of the 5 µm filter were immersed in 900 µL of 40% formamide hybridization buffer (40% deionized formamide, 10% dextran sulfate, 900 mM NaCl, 20 mM Tris-HCl pH 7.5, 0.01% sodium dodecylsulfate (SDS), 1% Blocking agent from Boehringer Mannheim) and 3 µL of oligonucleotide probe (50 ng µL^−1^ stock solution, final probe concentration 0.17 ng µL^−1^). Hybridization was performed at 35°C for 2–3 hours in a 1.5 mL microcentrifuge tube temperature block (Eppendorf NA). Filters were washed twice at 37°C for 5 min with 25 mL of fresh washing buffer. Each section was incubated in Tris-NaCl-Tween (TNT) buffer for 15 min at room temperature in the dark. Tyramide reactions were carried out using a TSA kit (NEL741001KT TSA Plus Fluorescein System, PerkinElmer); 30 µL of freshly made TSA mix was applied to cover each filter. Next, slides were incubated for 15 min at room temperature, then transferred to TNT buffer baths for 15 min.

Dinospore abundances were estimated following Siano et al., [13]. In brief, one eighth sections of the 0.8 µm filters were covered by 30 µL of hybridization buffer and 3 µl of oligonucleotide probe (50 ng µL stock solution) and incubated at 42°C for 3 h. Filters were then washed twice at 47°C for 30 min each. After the TSA reaction, sections were washed twice in 55°C pre-warmed TNT baths for 30 min. After hybridization, all filters were counterstained and mounted as described by Alves de Souza et al., [26]. Slides were stored at 4°C in the dark for a week before analysis. All hybridized and stained filters were observed with a Zeiss Axioskop epifluorescence microscope equipped with 40X and 63X objectives coupled with fluorescence filter sets for observing calcofluor, propidium iodide (PI) and fluorescein tyramide staining.

**Figure 2:**
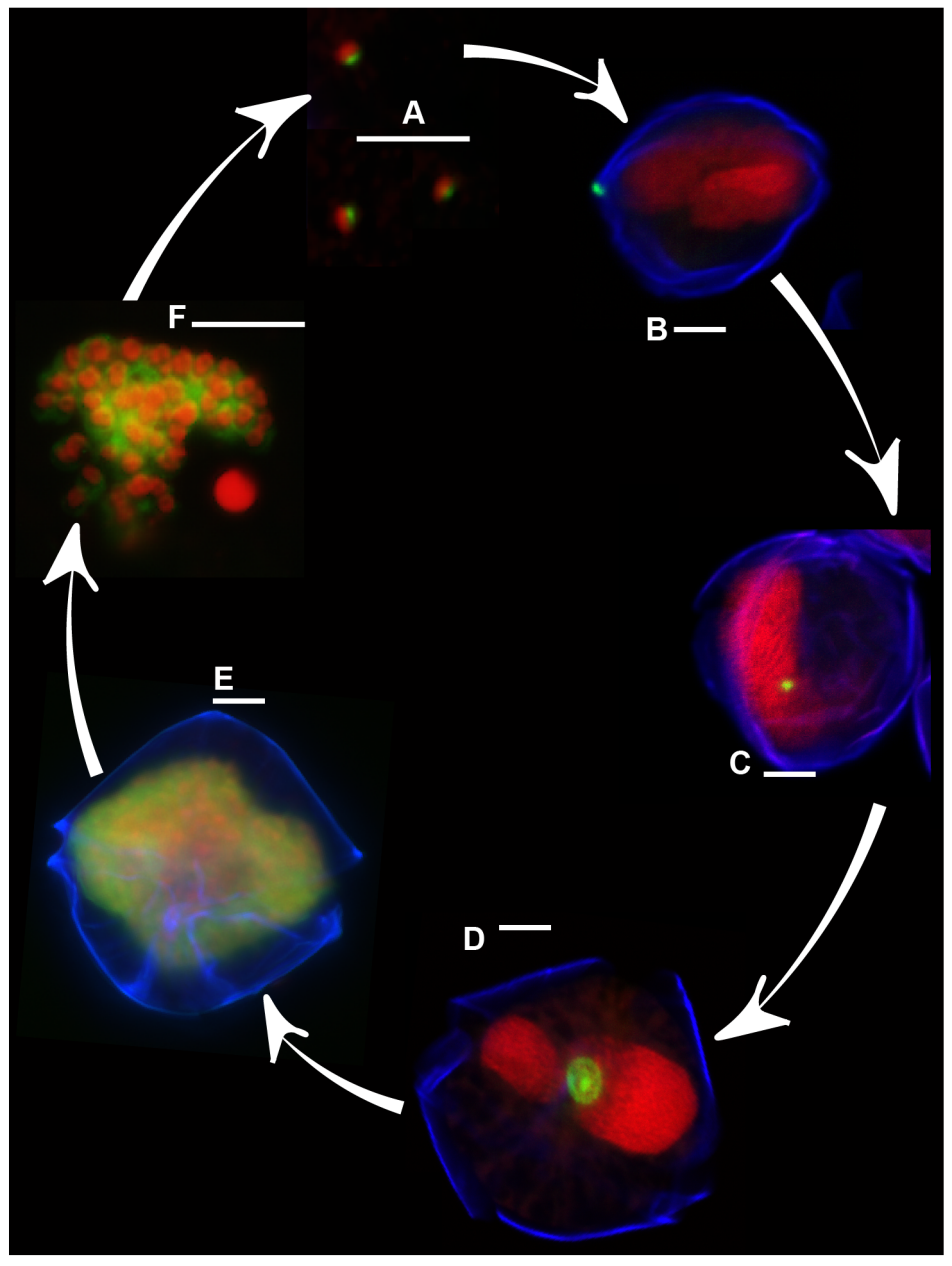
Different stages of *Amoebophrya* sp. infecting *A. fundyense* in Salt Pond as revealed by FISH-TSA. Green fluorescence shows the ALV01 probe targeting on the parasite. Red and blue fluorescence mark the host nucleus and theca respectively. (A) Dinospores, (B) initiation of the infection, dinospore attached to the host theca, (C) early, (D) intermediate and (E) beehive stages of infection (F) free-living vermiform. Scale bars = 10 µm.

All infected *A. fundyense* on a filter section were counted. *A. fundyense* cells were considered infected when the nucleus of the cell and the ALV01 probe signal were both detected on the surface or inside the host. Infection prevalence was estimated when at least 400 *A. fundyense* host cells were observed (confidence limit at 95% significance level (+/−, 10%). Infected host cells were categorized into three groups according to the development of the *Amoebophrya* trophont (1) ‘early’ if the parasitoid was present as a small dot attached to the host theca or nucleus (Fig. 2B and Fig. 2C); (2) ‘intermediate’ if the parasitoid occupied a large portion of the host nucleus (Fig. 2D); and (3) ‘mature’ if a trophont was present (i.e. the “beehive” stage of infection; Fig. 2E).

Host mortality induced by *Amoebophrya* spp., the percentage of hosts killed per day, was estimated following a modification of an equation from Coats & Park [8] :
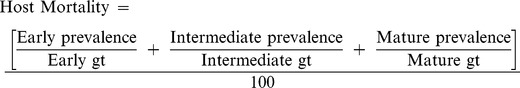
where generation times for early, intermediate and mature stages were estimated to be 3.5, 2.5, and 1 day for early, intermediate and late-stage infections respectively. These maturation time estimates are based on a culture experiment (data not presented here).

*Amoebophrya* dinospores were counted with a 63X objective in either 20 randomly chosen fields or on the whole surface of the analyzed piece of filter depending on dinospore density. If less than 75 dinospores were found in 20 fields, filter sections were fully scanned. *Amoebophrya* spp. dinospore concentration was estimated when at least 400 free-living *Amoebophrya* cells were observed (confidence limit at 95% significance level (+/−, 10%).

### 2.5 Observations of A. fundyense Life-cycle Stages

*A. fundyense* life-cycle stages were enumerated on FISH-TSA stained filters. A minimum of 200 *A. fundyense* cells per sample was observed at 40X to calculate planozygote percentages and concentration (confidence limit at 95% significance level (+/−, 14%). Planozygotes were identified based on cell size (>45 µm diameter) and morphology as previously described by Anderson et al. [28] and Brosnahan et al. [29]. Examples of mating *A. fundyense* gamete pairs of *A. fundyense* cells were distinguished from dividing cells by their angle of attachment. Whereas dividing *A. fundyense* are always oriented relative to one another with their cingular grooves parallel [30], the cingular grooves of fusing gametes are often oriented obliquely, allowing them to be distinguished after fixation and staining.

## Results

### 3.1 A. Fundyense Distribution in Salt Pond

**Figure 3:**
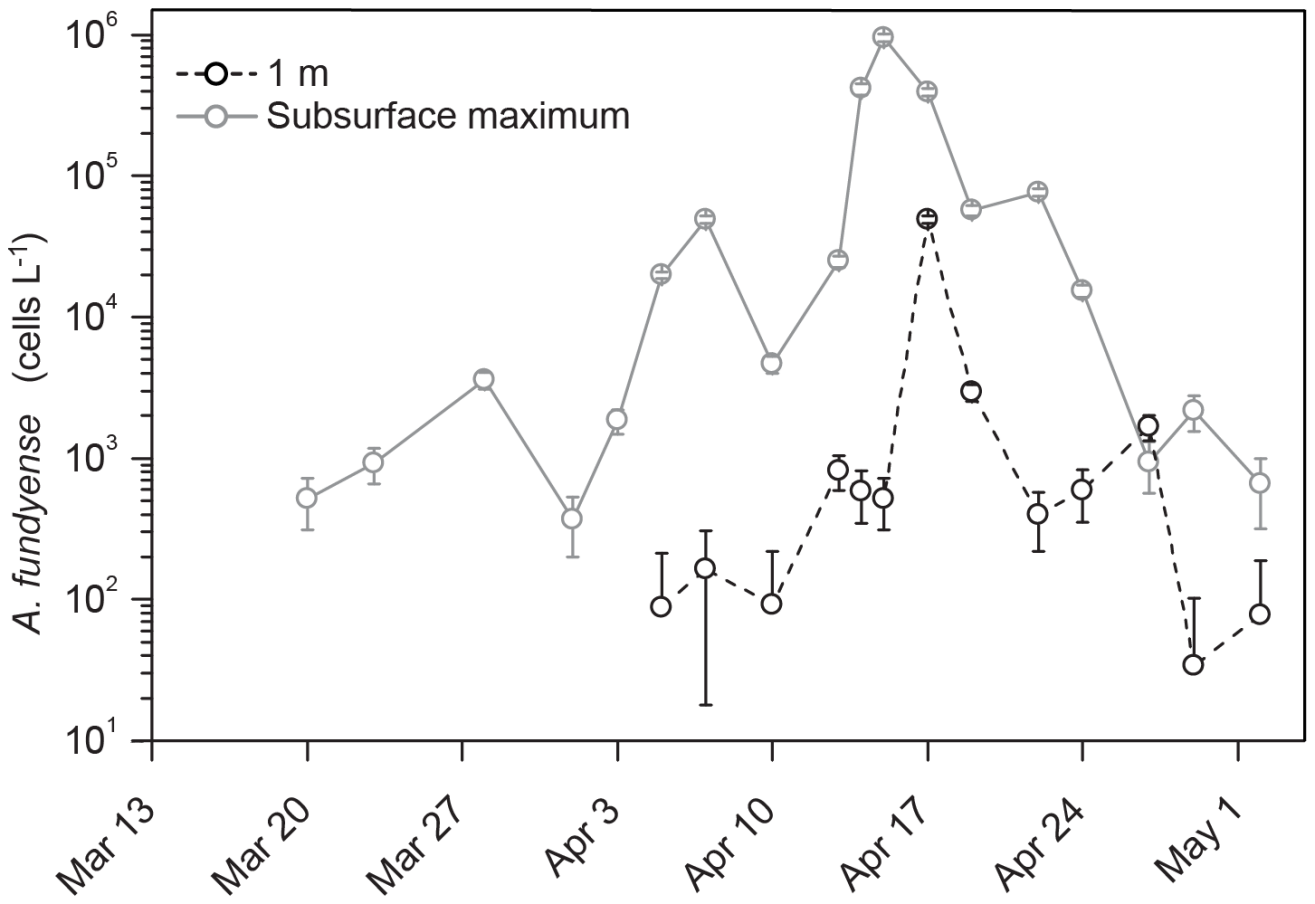
Salt Pond *A. fundyense* vertical distribution from March to May 2012. Subsurface maximum was obtained from the highest concentration – either from 3 or 5 m depth - as the most representative sample of the *A. fundyense* population for each sampling day.

The maximum concentration of *A. fundyense* cells was always between 3 and 5 m depth throughout the 2012 bloom season, a pattern that reflected the diel vertical migration behavior of this organism. Because the sampling times were not synchronized with this diel migration cycle, the highest concentration obtained – either from 3 or 5 m depth - was chosen as the most representative sample of the *A. fundyense* population for each sampling day. At the start of the sampling period (20 March), the *A. fundyense* concentration was greater than 100 cells L^−1^ at the subsurface maximum in Salt Pond, with no cells observed in surface waters (Fig. 3). By April 5 and 7, *A. fundyense* concentrations had increased to greater than 100,000 cells L^−1^ and one week later on April 15, the population had increased to ∼1×10^6^ cells L^−1^. Concentrations of *A. fundyense* remained high for 4 days (April 14–17), but then decreased more than 10-fold (from 390,231 to 10,180 cells L^−1^) over the next week (April 19–24). *A. fundyense* concentrations remained at less than 1,000 cells L^−1^ from April 24 until the end of the study (May 04). Throughout the development phase of the *A. fundyense* bloom, surface cell concentrations remained low, especially in comparison to the sub-surface where concentrations were typically 10–100 -fold higher. A single exception to this pattern was April 27 when the observed *A. fundyense* concentration at the surface was less than 2-fold higher than at 3 m, the subsurface maximum on that day.

### 3.2 A. fundyense Life Cycle Stages in the Water Column

**Figure 4:**
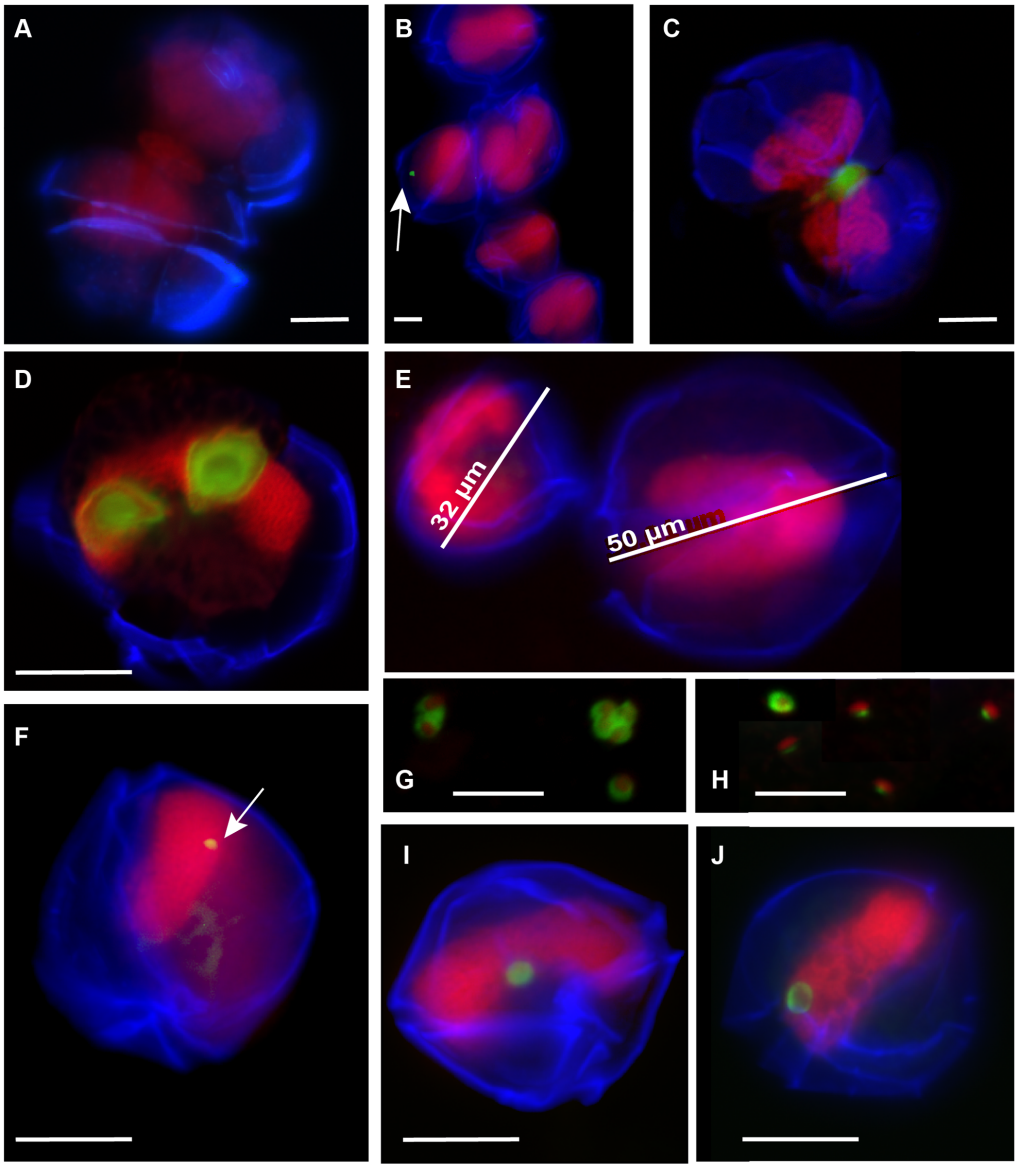
Micrographs of *A. fundyense* and *Amoebophrya* spp. life-cycle stages as detected by FISH-TSA. Green fluorescence shows the ALV01 probe targeting on the parasite. Red and blue fluorescence mark the host nucleus and theca respectively. (A) Non infected fusing gametes; (B) *A. fundyense* chain showing one dinospore attached to a recently divided cell (white arrow); (C) infected *A. fundyense* gametes undergoing conjugation. (D) *A. fundyense* planozygote showing double infection with incipient mastigocoels. (E) Differences between a vegetative cell (32 µm diameter) and a planozygote (50 µm). (G) 2–4 clusters and (F) individual dinospores. Early infected planozygotes (F–J). Scale bars = 10 µm.

**Figure 5:**
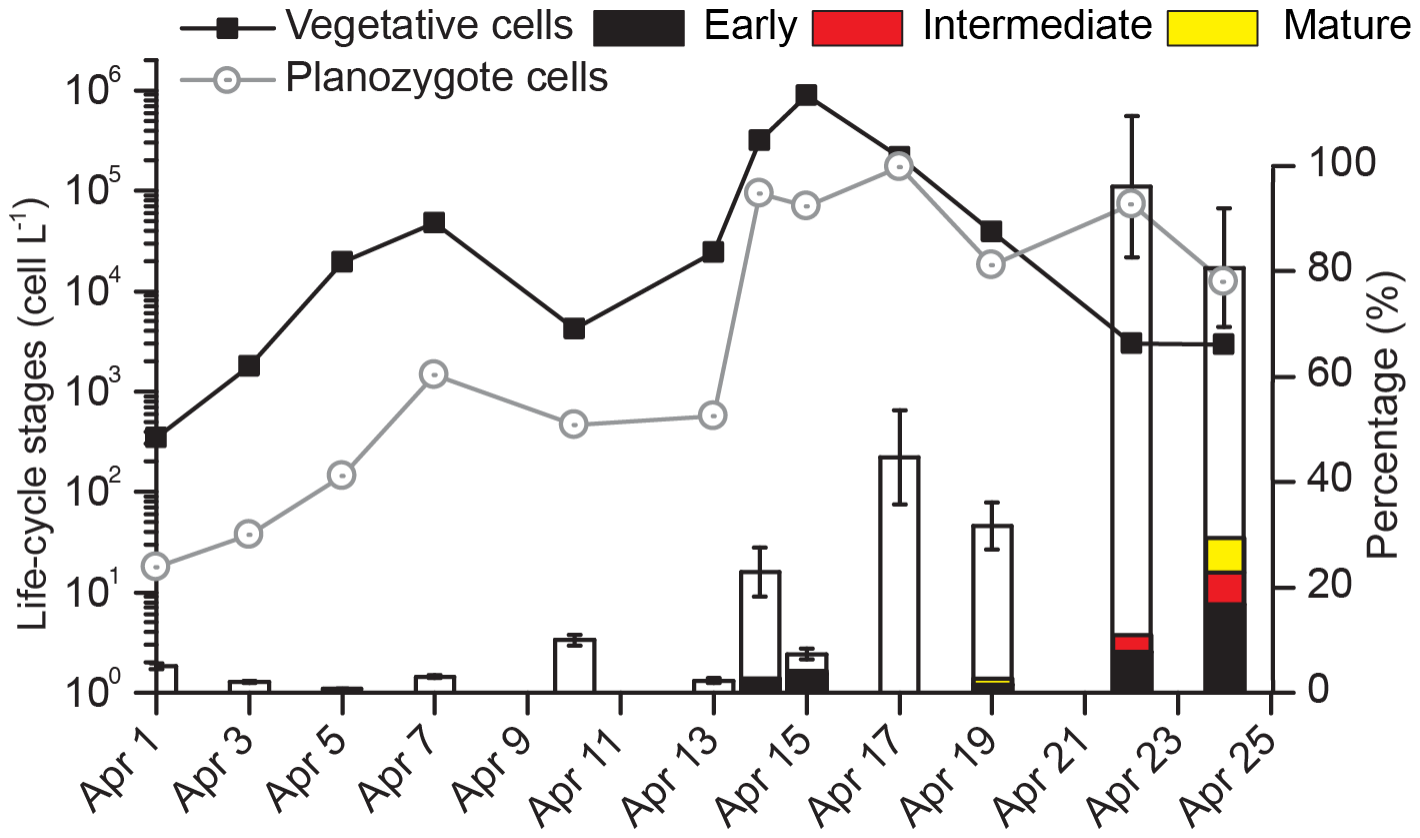
Time-series of *A. fundyense* life-cycle stages at the subsurface maximum from 1 to 24 April. Vegetative cells (black squares), planozygotes (>45 µm diameter, grey open circles), and percentages of planozygotes (white bars) relative to the total *A. fundyense* population. *Amoebophrya* spp. infections and their maturation stage (%; black, red and yellow bars) are overlapped to the percentage of total planozygote abundance.

Planozygotes (defined here as large cells greater than 45 µm; Fig. 4E) represented between 2.3% and 96% of the total planktonic *A. fundyense* over the course of the bloom (Fig. 5). The lowest planozygote percentages were observed between April 3 and13. Following the peak (April 16), there was a significant increase in planozygote abundance with the maximum coinciding with the end of the bloom (April 22–24; Fig. 5). From April 15 to 19– just a few days prior to the bloom’s termination – a high proportion of fusing gametes (Fig. 4A) was observed in filtered samples (data not shown). These results are in agreement with a near continuous life-cycle analysis performed during the same time window with an Imaging FlowCytobot (IFCB) [21] that was deployed at 5 m depth below the float where water samples were taken for this study [22]. IFCB monitoring showed a rapid transformation to low volume cells (presumably gametes) on April 16 and a rapid decline in cell division (observed through a downward shift in the frequency of doublet vegetative cells; data not shown).

### 3.3 Infections by Amoebophrydae Parasitoids

The Amoebophrydae-specific probe ALV01 revealed that infections were restricted to just *A. fundyense* in Salt Pond in 2012. No infections were observed in any other potential dinoflagellate hosts present in the pond (*Dinophysis* spp., *Gonyaulax* spp., *Neoceratium* spp. among others). Multiple co-infecting dinospores (up to 5) were detected in some *A. fundyense* hosts (Fig. 4D), but only one vermiform was observed within mature parasitized hosts. FISH-TSA sensitivity allowed detection of all life-cycle stages of *Amoebophrya* spp., including very early infections (dinospores attached to their host surface; Fig. 2B and Fig. 4B) and free-living dinospores (∼5 µm equivalent spherical diameter, Fig. 2A and Fig. 4G–H). Asexually dividing cells were never found to contain parasites inside their nuclei. Conversely, very early *Amoebophrya* spp. infections - not yet associated with the cell nucleus - were observed in recently divided cells forming 2- and 4-cell chains (Fig. 4B). Infected gametes were observed to fuse with *Amoebophrya* spp. parasite inside at least one of their nuclei (Fig. 5C). Early infections were observed (up to 20%) in planozygotes after 19 Apr (Fig. 5 and Figs 4F–J).

**Figure 6:**
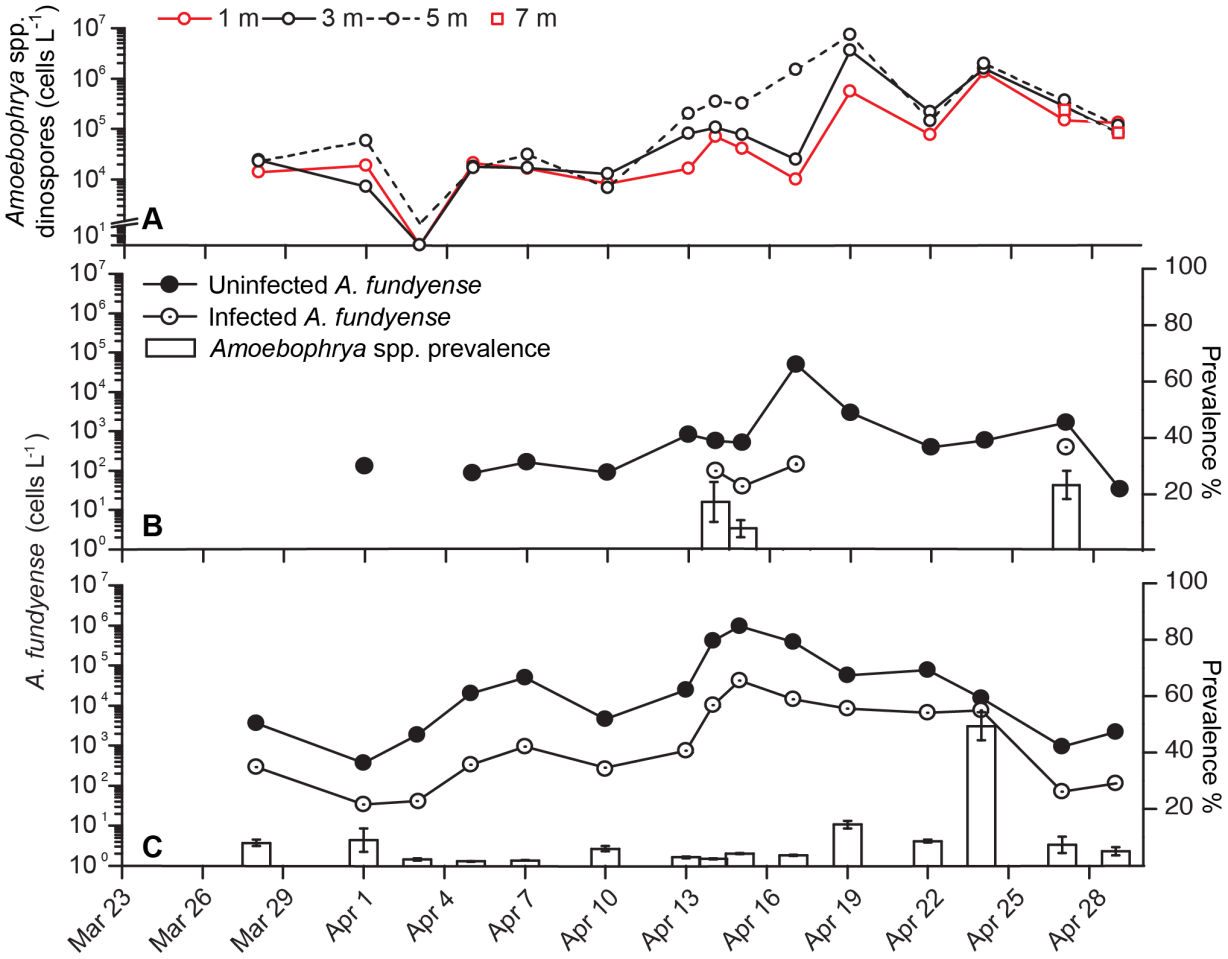
Vertical and temporal dynamics of *Amoebophrya* spp. dinospores and *A. fundyense* infections. *Top panel (A)*. *Amoebophrya* spp. dinospore concentrations observed at 1 (red solid line), 3 (black solid line), 5 (black dotted line) and 7 (square dots) meters depth. *Bottom panels (B and C)*. Uninfected (black dots-black line) and infected (white dots-black line) *A. fundyense* cell concentrations (cell L^−1^) and *Amoebophrya* spp. prevalence (%; white bars) at 1 m (B) and the subsurface maximum (C) at Salt Pond, station 21, from 24 March to 5 May 2012.

Infected *A. fundyense* cells were observed in the subsurface maximum throughout most of the study period (Fig. 6C). Beginning April 1, the number of infected *A. fundyense* cells increased steadily until April 15 when the overall *A. fundyense* concentration also reached its peak. Although the number of infected cells reached ∼50,000 cells L^−1^, the proportion of *A. fundyense* hosts infected by *Amoebophrya* spp. remained low (<5%). The proportion of infected hosts increased over the next week (April 16–23) as the *A. fundyense* host population declined. Highest proportions of hosts infected were observed on April 24 (50%), but the overall abundance of infected cells remained steady between 5,000 and 15,000 cells L^−1^. Infected cells were always scarce at the surface (1 m depth; Fig. 6B).

**Figure 7:**
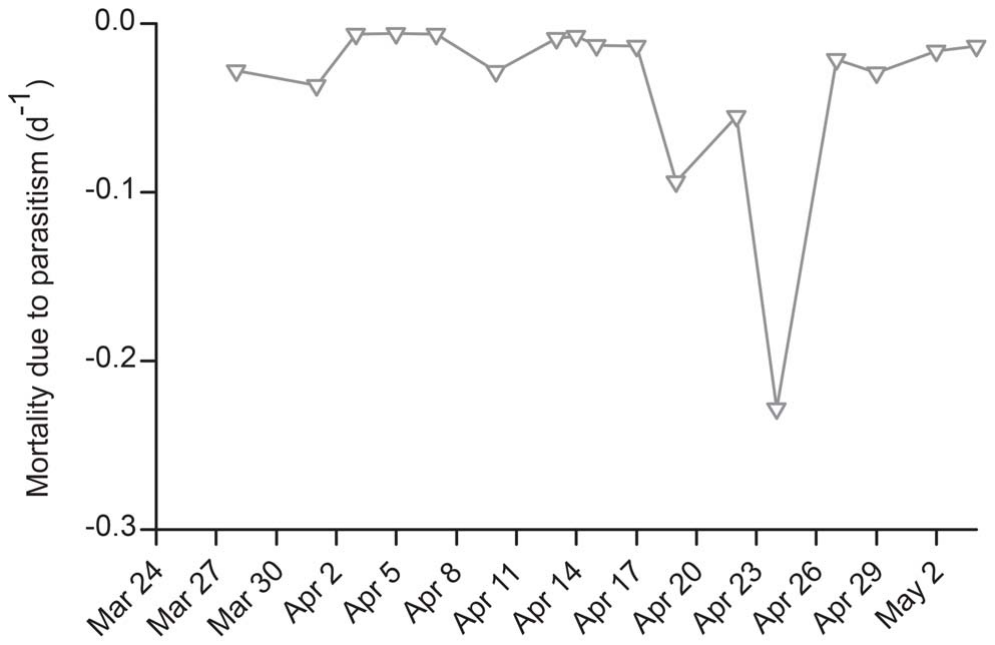
Estimates of mortality rates of the *A. fundyense* population due to parasitism by *Amoebophrya*. Open triangles-grey line show the evolution of *A. fundyense* mortality due to *Amoebophrya* spp. parasitism (d^−1^) 30 March to 2 May at the subsurface maximum (3–5 m depth).

The estimated host mortalities due to *Amoebophrya* spp. infections at the subsurface maximum (3–5 m) during the peak on April 15 were −0.013 d^−1^ (Fig. 7). Assuming that all infected cells eventually died, infections killed less than 1% of the *A. fundyense* total population. The mortality rate due to parasitism subsequently increased from −0.013 d^−1^ April 17 to −0.09 April 19 and a maximum estimated rate of −0.23 d^−1^ April 24. These rates compare to a mean apparent population loss rates of −0.65 d^−1^ over the same time period. The difference in these estimates likely reflects losses due to other factors, including grazers and especially to sexual encystment. It is also important to note that each of these estimates likely reflects error associated with spatial patchiness in the distribution of cells and parasites in the pond. This is a potentially significant shortcoming of inferring rates from time-series of concentration in natural systems, even those like Salt Pond where advection by the mean flow is unlikely to have a significant impact. Despite the fact that spatial variability is a potentially significant source of error, the inferred rates are nevertheless valuable for assessing the impact of parasitism.

**Figure 8:**
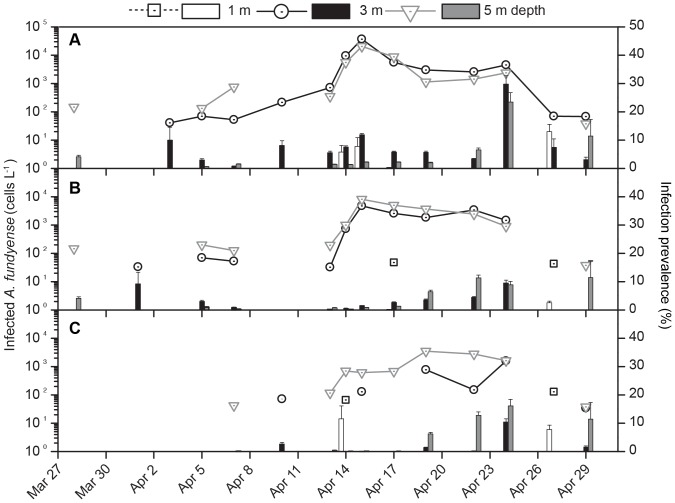
Vertical distribution of *Amoebophrya* spp. maturation stages in infected *A. fundyense* cells. Early (A), intermediate (B) and beehive (C) infected cells (scatter-lines; cell L^−1^) and prevalences (bars; %) of *Amoebophrya* spp. Infections at 1 m (open squares- dotted black lines and white bars), 3 m (open circles- solid black lines and black bars) and 5 m (open triangles-solid grey lines and grey bars) depth during the sampling season (27 March to 01 May).

The maturation states of *Amoebophrya* spp. infections were considered when calculating general infection rates (Fig. 8). Early infections were more frequent at 3 m depth, and found throughout the entire time-series whereas intermediate and mature infections were usually found deeper (5 m) and - especially in the case of beehive stages - later in the season (from April 13 to 24).

*Amoebophrya* dinospores were present throughout the sampling period (March 28 to May 2; Fig. 6A) in concentrations that varied from 10,000 cells L^−1^ early in the sampling season (March 28) to <1,000 cells L^−1^ during surveys conducted through April 3 and up to 8×10^6^ cells L^−1^ after the peak of the bloom on April 19^th^ (Fig. 6A). This final, dramatic increase in dinospore abundance followed an increase in the number of infected *A. fundyense* hosts during the peak in *A. fundyense* abundance 3–4 days earlier (April 14–15; Fig. 6). A second spike in dinospore abundance was observed 5 days later on 24 Apr (∼ 1×10^6^ cells L^−1^). Dinospore numbers then decreased steadily to level below the limit of detection (<100 cells L^−1^) after the April 28.

Dinospore appearance was different between the two main peaks in abundance. On April 19, vermiform structures (Fig. 2G) and dinospores clusters of 4 or 2 nuclei were abundant (Fig. 4G; data not shown). Individual dinospores were round, big and contained a large cytosol that surrounded a dense nucleus. In contrast, dinospores found on 24 Apr were smaller, less round and contained little cytoplasm (Fig. 4H). No vermiform or cluster structures were found at the end of the bloom.

## Discussion

*In situ* observations [9], [11], [26] and modeling studies [15], [31] have suggested that *Amoebophrya* parasites can control and even prevent harmful algal blooms. However, host-parasite dynamics and their role in HAB termination in the field are not yet well understood. This study adds significantly to our knowledge of the *Amoebophrya* spp.-host dynamics in the field and contributes to our understanding of the quantitative importance of parasites to HAB termination that may be applicable to restricted embayments worldwide.

The *A. fundyense*-*Amoebophrya* dynamic in 2012 in Salt Pond shared some characteristics with observations of host-*Amoebophrya* spp. in other systems [11], [26], [32]. Peaks in *A. fundyense* cell concentrations were followed by increased infection levels and subsequently, by release of dinospores 4–5 days later. High concentrations of free-living dinospores were brief, as the swimming parasites must infect new hosts or they die within 3–4 days, based on the high mortality rate observed under culture conditions [7].

However, mortality due to *Amoebophrya* spp. infections was not responsible for the *A. fundyense* bloom decline, as a loss of 1% per day of the *A. fundyense* cells to parasitism could not have offset the increase of *A. fundyense* population during the early development of the bloom.

Dinospore: host ratios during the main two dinospores peak releases were as high as 200∶1 but infection prevalence never exceeded 55%. High dinospore inoculation (20∶1, 40∶1, 115∶1) in culture has been shown to result in significant high infections (up to 90%; [7]). However, on average, *Amoebophrya* spp. infected and killed ∼30% per day of the already declining *A. fundyense* population in the end phase of the bloom. Dinospore concentrations reported from natural systems have never been higher than 10^5^–10^6^ mL^−1^ nor the parasite-host ratio higher than 2∶1 [11], [13], [26]. Nevertheless, this is the first report of *Amoebophrya* spp. dinospore abundance as high as ∼8×10^6^ cells L^−1^ (April 19, 5 m depth, Fig. 6A) in the field.

Many factors can affect infections in natural populations, such as host concentration; encounter rates, and genotypic variability within the host. Field and modeling studies have also shown that grazing by ciliates can limit the success of *Amoebophrya* spp. dinospores, with differential grazing pressure potentially influencing parasite prevalence [15], [33]. This process is called the ‘dilution effect’ and has been reported for many different parasite-host systems [34].

Sexual processes [35] and life-history shifts (e.g. the Cheshire cat strategy, [36]) have been pointed out as host refuge strategies to avoid parasite and viral infections. Dinoflagellates have been shown to stop dividing in culture once infected by *Amoebophrya* spp. [32] a result that is consistent with our observations in Salt Pond since no infections were found inside the nuclei of asexually diving *A. fundyense* pairs. However, we did observe the fusion of infected *A. fundyense* gametes (Fig. 4B and Fig. 4C) in the field suggesting that *Amoebophrya* spp. infections do not prevent sexual processes in their host populations. Based on comparison with culture experiments of trophont development, the size and location of trophonts found inside fusing cells (Fig. 4B) suggest that infection occurred at least 48 hours before fusion. However, it was not possible to document the fate of these fusing gametes, nor whether *Amoebophrya* spp. infections might have arrested gamete fusion/zygote development afterwards. Early trophonts were frequently observed in planozygotes (Fig. 5 and Fig. 4F–J) at the end of the bloom, suggesting that new dinospore recruitment, and not inherited gamete infection, was the main *Amoebophrya* spp. infection pathway for zygotic cells.

Although considerable scientific effort has been devoted to understand the effect of life-cycle strategies on the decline of HABs, questions regarding the role of biological factors causing the induction of sexuality remain unresolved. While most of the observed life-history shifts in dinoflagellate populations have been related to chemical factors [37], biological factors may also play an important role [35], [38]. Past studies at Salt Pond have reported the formation of *A. fundyense* planozygotes under favorable environmental conditions for cell growth with no obvious induction from physico-chemical factors [28]. Maximum values of planozygote percentages for *A. fundyense* observed by these authors were 35–40%. In 2012, planozygotes were present in low concentrations during the logarithmic growth phase of the bloom (14 Apr; Fig. 5). Yet, planozygote percentages reached very high proportions during the final stages of bloom termination. An apparent life-cycle shift occurred on April 15 in Salt Pond in 2012, and was already evident before the main release of dinospores on April 19. This fact suggests that the observed shifts (gamete formation, fusion, planozygote formation and encystment) during the *A. fundyense* bloom in Salt Pond is unlikely to be related to a high abundances of *Amoebophrya* spp. free-living dinospores (April 19), contrary to suggestions by several authors working in other systems [39], [40]. However, the increase in the number of parasitized *A. fundyense* cells at the time of the host peak (April 14) does suggest a possible trigger for sexual synchronization of *A. fundyense* populations in 2012. This hypothesis poses interesting questions. Can chemical cues such as water-borne signals or cell-to-cell interactions induce sexual changes in healthy *A. fundyense* cells? Similar findings have been recently reported for the coccolithophorid *Emiliania huxleyi* viral infections and could be compared with “quorum sensing” in bacterial biofilms [41]. Strongly supporting this hypothesis is the dramatic sexual response of *A. fundyense* cells. Further studies are needed to demonstrate if *A. fundyense* cells are capable of producing, sensing and responding to their own parasite-induced stress signals in both culture and field studies.

*Amoebophrya* spp. infected hosts have been observed to exhibit distinct swimming behavior and physiological patterns relative to healthy cells [32], [42]. Coats and Bockstahler [8] reported a sharp vertical separation of infected and uninfected hosts in the field, and detected a high percentage of mature infections several meters below the lightly infected surface population. Our results agree with these field [8] and culture [42] observations. In our study, maturation stages of *Amoebophrya* spp. infections also displayed vertical zonation (Fig. 8). Early infections were found in the upper 3 m while beehives were more common at 5 m depth. *A. fundyense* cells are known to follow a specific diel vertical migration in Salt Pond and are selectively retained due to general avoidance of surface layers [20]. However, infections by *Amoebophrya spp.* may interrupt internal mechanisms that control the vertical migration of their hosts.

To understand the quantitative effect of *Amoebophrya* spp. on host populations, it is important to recognize all parasite life stages. Newly formed *Amoebophrya* dinospores quickly lose their capacity to infect and most die after 2 to 10 days in cultures lacking hosts [7]. Furthermore, the mechanism that allows *Amoebophrya* spp. to survive during the dormancy period of their hosts remained an open question for many years. Recently, Chambouvet et al., [39] demonstrated the ability of some *Amoebophrya* strains to lie dormant inside the cyst of its host (*Scrippsiella trochoidea)*. If this finding is generalizable, it would explain how *Amoebophrya* spp. can specifically infect the same host species year after year in natural environments like Salt Pond, even though the host has a meroplanktonic lifestyle and is present for most of the year in its cyst form, which is resistant to new infections [39]. A recent modeling study used a simplified representation of this shared-cyst survival strategy to understand parasite-host dynamic in close environments [43]. The authors concluded that when both parasites and hosts emerge from cysts through germination, hosts could reach high concentrations before becoming heavily infected. The intensity of the host bloom will depend on the number of parasitized cysts in the cyst beds. However, parasites would take over and the host population would be eliminated eventually unless the host population has a counter-balancing refuge strategy. Although *A. fundyense* blooms in Salt Pond are known to originate from germination from local cyst beds [23], there is still no evidence of a shared-cyst link between *Amoebophrya* and *Alexandrium* spp. Further work is needed to establish whether *A. fundyense* cysts provide a refuge for local *Amoebophrya* spp. populations.

Large numbers of dinospores were produced during the development and decline of the *A. fundyense* bloom in Salt Pond. Nevertheless, *Amoebophrya* spp. did not infect any other dinoflagellate species present. This strongly suggests that *Amoebophrya* spp. is host-specific for *A. fundyense* in our study area. Our results agree with the observations made by Chambouvet et al. [11] in the Penzé estuary (France). Those authors found highly host-specific interactions with a single *Amoebophrya* clade infecting a single dinoflagellate host throughout several years. However, isolated strains of *Amoebophrya* spp. from Salt Pond have been able to successfully infect different species of dinoflagellates (*Alexandrium*, *Prorocentrum, Scrippsiella,* and *Heterocapsa*) in culture [44], [45], suggesting that at least some *Amoebophrya* strain in Salt Pond are able to infect a wide range of hosts [3]. These conclusions, however, are based on culture observations alone and *Amoebophrya* species may have a narrower host range in the field.

Understanding host-*Amoebophrya* interactions is a high priority research topic with implications for HAB control and prevention. Host-parasite interactions are characterized by an ‘arms race’ that results in the fast co-evolution between both populations (the Red Queen hypothesis, [46]). The Red Queen hypothesis [47] explains how pathogens may maintain sexual reproduction in hosts and suggests that parasites have a great impact on driving host biodiversity. *A. fundyense* blooms in Salt Pond have been characterized by extensive genetic diversity and rapid temporal genetic differentiation [48]. *Amoebophrya* infections were suggested by these authors as one of the underlying reasons for these genetic differences. Results from our study provide direct, empirical evidence that *Amoebophrya* specifically infect *A. fundyense* in Salt Pond, suggesting that parasitism may control the genetic structure of the pond’s *A. fundyense* population.
